# Stem Cells and Their Derivatives—Implications for Alveolar Bone Regeneration: A Comprehensive Review

**DOI:** 10.3390/ijms222111746

**Published:** 2021-10-29

**Authors:** Dušan Hollý, Martin Klein, Merita Mazreku, Radoslav Zamborský, Štefan Polák, Ľuboš Danišovič, Mária Csöbönyeiová

**Affiliations:** 1 Department of Stomatology and Maxillofacial Surgery, Faculty of Medicine, Comenius University, Heydukova 10, 812 50 Bratislava, Slovakia; info@hollydent.sk (D.H.); merita.mazreku@gmail.com (M.M.); 2 Institute of Histology and Embryology, Faculty of Medicine, Comenius University, Sasinkova 4, 811 08 Bratislava, Slovakia; martin.klein@fmed.uniba.sk (M.K.); stefan.polak@fmed.uniba.sk (Š.P.); 3 National Institute of Rheumatic Diseases, Nábrežie I. Krasku 4, 921 12 Piešťany, Slovakia; info@drzamborsky.sk (R.Z.); lubos.danisovic@fmed.uniba.sk (Ľ.D.); 4 Department of Orthopedics, Faculty of Medicine, Comenius University and National Institute of Children’s Diseases, Limbová 1, 833 40 Bratislava, Slovakia; 5 Institute of Medical Biology, Genetics and Clinical Genetics, Faculty of Medicine, Comenius University, Sasinkova 4, 811 08 Bratislava, Slovakia

**Keywords:** alveolar bone regeneration, stem cell-based therapy, tissue engineering, exosomes

## Abstract

Oral and craniofacial bone defects caused by congenital disease or trauma are widespread. In the case of severe alveolar bone defect, autologous bone grafting has been considered a “gold standard”; however, the procedure has several disadvantages, including limited supply, resorption, donor site morbidity, deformity, infection, and bone graft rejection. In the last few decades, bone tissue engineering combined with stem cell-based therapy may represent a possible alternative to current bone augmentation techniques. The number of studies investigating different cell-based bone tissue engineering methods to reconstruct alveolar bone damage is rapidly rising. As an interdisciplinary field, bone tissue engineering combines the use of osteogenic cells (stem cells/progenitor cells), bioactive molecules, and biocompatible scaffolds, whereas stem cells play a pivotal role. Therefore, our work highlights the osteogenic potential of various dental tissue-derived stem cells and induced pluripotent stem cells (iPSCs), the progress in differentiation techniques of iPSCs into osteoprogenitor cells, and the efforts that have been made to fabricate the most suitable and biocompatible scaffold material with osteoinductive properties for successful bone graft generation. Moreover, we discuss the application of stem cell-derived exosomes as a compelling new form of “stem-cell free” therapy.

## 1. Introduction

The restoration of severe periodontal defects, such as damage to the alveolar bone or soft periodontal tissue, is still a complex and challenging field for clinicians. There are various causes of bone defects including congenital anomalies, medications, local inflammation, periodontitis, traumatic injuries, malignancies, and dental surgical interventions. The traditional therapy to overcome bone atrophy relies on the use of more or less invasive techniques. Autologous alveolar bone grafts represent “the gold standard” due to their osteogenic, osteoinductive, and osteoconductive properties. Therefore, they are the first-choice option in the reconstruction of large bone defects [1]. However, the use of autografts is usually associated with donor site morbidity, graft failure, and immunological rejection. A limited source of graft tissue is also a problem that needs to be addressed. Moreover, this procedure is painful and often results in prolonged hospitalization. There are some other bone tissue substitutes, such as allografts and xenografts; however, their disadvantages include the possibility of immune rejection and pathogen transmission from the donor to the host. The application of synthetic grafts is also limited because of their non-optimal integration with native tissue, which can often lead to graft failure [2,3,4]. As of today, an ideal technique with the ability to completely regenerate harmed bone tissue has not been found.

**Figure 1:**
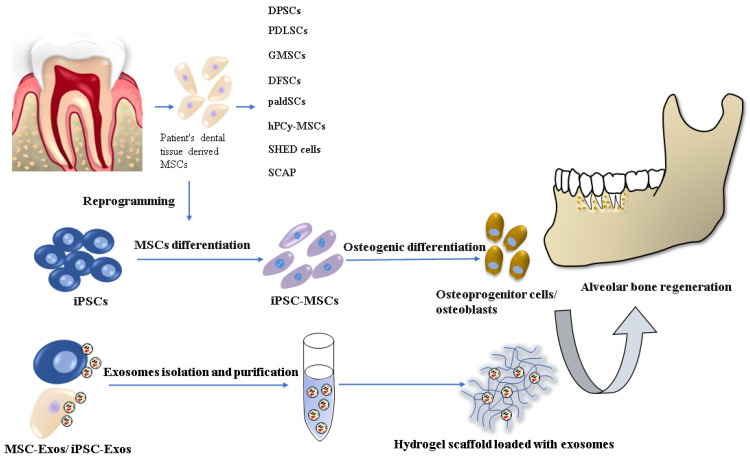
Stem cell-based tissue engineering methods for alveolar bone regeneration.

A promising alternative in reconstructing alveolar bone tissue is tissue engineering techniques and stem cell-based regenerative therapies, where the key factor is the most suitable combination of cells, scaffolds, and signaling molecules [5] (Figure 1).

There are already several dentistry regenerative approaches based on the most commonly used stem cell type: mesenchymal stem cells/stromal cells (MSCs), which have been applied in implantology and periodontology. MSCs, with their multilineage differentiation potential (differentiation into osteocytes, chondrocytes, adipocytes, muscle cells, and even neurocytes) are widely available from various tissues sources. The MSCs used in oral and maxillofacial regions are usually harvested from bone marrow, adipose tissue, and dental tissue [6,7,8]. However, their cultivation and expansion are time-consuming, resulting in a senescent cell population. Other cell lineages, which has been used in in vitro and in vivo animal studies are embryonal stem cells (ESCs) and induced pluripotent stem cells (iPSCs). Nevertheless, the use of ESCs is related to serious ethical concerns involving the controversial in vitro human blastocyst destruction. On the other hand, iPSC research promises great potential for dental tissue regeneration thanks to its similar characteristics to ESCs but with no ethical issues. Precisely, iPSCs can be generated from several adult somatic cells specific for the concrete patient. Despite the many advantages of iPSC technology, there are still several safety challenges, such as teratoma formation or malignant transformation, which have to be solved before their clinical application [9,10].

## 2. Osteogenic Potential of Dental Tissue-Derived MSCs

The regenerative process of alveolar bone reconstruction is based on ossification during embryonal development, where the osteoblasts differentiated from MSCs produce osteoid, an unmineralized bone matrix, with bone mineralization following. Moreover, MSCs with their paracrine secretion of cytokines and growth factors are also able to enhance bone regeneration indirectly. Released factors, such as tumor necrosis factor-*α* (TNF-*α*), platelet-derived growth factor (PDGF), interleukin-1 (IL-1), and IL-6, can initiate further activation of MSCs and their recruitment in regenerated sites [11,12]. Therefore, successful bone tissue engineering should involve a combination of abundant MSCs/ osteoprogenitor cells, a suitable mixture of biofactors to induce osteogenic differentiation, and scaffolds based on biomaterials. For dental tissue regeneration, the most eligible are MSCs derived from various dental sources, such as dental pulp—dental pulp stem cells (DPSCs) [13]; periodontal ligament—periodontal ligament stem cells (PDLSCs) [14,15]; gingiva—gingival mesenchymal stem cells (GMSCs) [16,17]; dental follicle or bilayered Hertwig’s epithelial root sheath—dental follicle stem cells (DFSCs) [18]; subepithelial palatal soft tissue—palatal-derived stem cells (paldSCs) [19,20]; periapical cyst tissue—human periapical cyst mesenchymal stem cells (hPCy-MSCs) [21,22]; and stem cells from exfoliated deciduous teeth (SHED cells) and from human root apical papilla (SCAP) [23] (Table 1).

Dental-derived MSCs display the same characteristics as bone marrow-derived MSCs (BM-MSCs); furthermore, they possess immunomodulatory and anti-inflammatory advantages in the local dental tissue environment [24].

The regenerative activity of MSCs following transplantation can be performed in several ways. It can be either by the direct engraftment and differentiation of MSCs into newly formed tissue or by immunomodulatory regulation of direct or indirect secretory signaling [25]. Bajestan et al. (2017) investigated the effectiveness of stem cell therapy for reconstructing alveolar cleft and trauma defects in adults. In a randomized controlled clinical trial, eighteen patients whose therapy was based on conventional autogenous block grafts or transplantation of autologous BM-MSCs processed by ixmyelocel-t were involved. Four months after transplantation, the grafting sites were re-entered to evaluate the implant stability, and in six months, the successfulness of the regenerative process was analyzed. In general, the results showed the capability of stem cell therapy to safely induce bone regeneration, nevertheless, with limited capacity in case of large alveolar defects [26].

DPSCs as a subpopulation of MSCs give rise to odontoblasts during tooth development. These cell populations have been the most studied dental stem cells for bone regeneration. The majority of studies reported the osteoinductive potential of DPSCs in vitro and in vivo [7,27,28]. Moreover, d’Aquino et al. (2007) found out for the first time that 30% of osteoblasts differentiated from DPSCs expressed not only osteocalcin but also specific antigens for endothelial cells, such as flk-1, CD54, von-Willebrand factor, CD31 (PECAM-1), and angiotensin-converting enzyme. Therefore, the authors transplanted DPSCs placed on bone chips into an in vivo model of immunocompromised rats. Interestingly, after 60 days, the formation of vascularized bone tissue was observed similar to adult bone, making them an ideal candidate for bone tissue replacement [29]. Tanikawa et al. (2020) performed a unique transplantation of deciduous DPSCs associated with hydroxyapatite-collagen sponges into the unilateral alveolar bone defect of six patients aged 8–12. After six months, during the post-operative evaluation, bone healing with no ectopic bone formation and graft loss was observed in all patients. However, the success of this first clinical trial using deciduous DPSCs was hindered by a small group of participants [30]. Paduano et al., 2021 demonstrated for the first time that the osteogenic capacity of DPSCs and DFSCs can be increased by their dedifferentiation into stem cell-like state (Dediff-DPSCs) under physiological conditions. Comparing the osteogenic potential between redifferentiated Dediff-DPSCs/DFSCs and osteogenic differentiated DPSCs/DFSCs revealed elevated expressions of Runx-2, osteocalcin, and osteonectin in redifferentiated DPSCs. Moreover, redifferentiated DPSCs/DFSCs exhibited higher formation of calcium nodules. Therefore, this study offers a new dedifferentiation approach to enhance the osteogenic potential of DPSCs/DFSCs without gene manipulation [31].

The osteogenic capacity of paldSCs in alveolar bone regeneration was proved by Grimm et al. (2014) in their clinical study with 30 patients. The authors combined allogeneic bone blocks with human adult paldSCs and implanted them into the alveolar bone defect. According to the performed analyses, the osteoinductive effect of paldSCs was manifested by improvement of vertical alveolar bone augmentation [19].

The DFSCs are tooth germ cells originating from the neural crest, which can be isolated from wisdom teeth. Due to their unique neuroectodermal origin, the DFSCs are direct precursors of periodontal tissues, such as periodontal ligaments, cementum, alveolar bone, as well as salivary gland cells [32]. Most in vitro studies proved the osteogenic properties of DFSCs in an appropriate osteoinductive medium [33,34,35]. However, there are only a few in vivo studies using murine or porcine models. Honda et al. (2011) used pellets with DFSCs to repair critical-sized calvarian defects in immunodeficient rats and observed bone formation similar to intramembranous ossification. The results of the immunohistological analyses showed the formation of a new bone matrix surrounded by osteoblasts [36].

GMSCs, quite novel postnatal stem cells, have attracted more attention during the past few years, thanks to their easy isolation, high proliferation capacity, and stable phenotype. Surprisingly, they can maintain telomerase activity in a prolonged culture with no tumorigenesis. According to the literature, GMSCs have high potential in the regeneration of alveolar bone defects, periodontium, oral neoplasms, and peri-implantitis [37,38,39]. To date, several studies have been published concerning the use of GMSCs in dental tissue regeneration. An interesting study was authored by Sun et al. (2019), who had systematically transplanted human GMSCs into a C57BL/6J mice model of severe periodontitis via the tail vein to observe their possible interaction with periodontal tissue. GFP-stained GMSCs were inserted in the second maxillary molar by a silk thread ligature. Four weeks post-transplantation, the histopathological analysis revealed significantly reduced alveolar bone loss, and the immunohistochemical staining detected GFP+ fibroblast-like cells and GFP+ osteoblasts within the area of newly formed alveolar bone [40]. In another recent study, Kandalam et al. (2021) investigated the bone regenerative capacity of pre-differentiated GMSCs combined with self-assembling hydrogel scaffold PuraMatrix™ and bone morphogenic protein (BMP2). The maxillary alveolar bone defect was surgically created in athymic rodent models and subsequently filled with GMSCs, which were pre-cultivated in an osteogenic medium for one week. The outcome was evaluated at 4- and 8-weeks post-implantation using microcomputed tomography and histological methods. In comparison with the control group, bone regeneration was significantly enhanced in groups who received pre-differentiated GMSCs treated with BMP2 and seeded on PuraMatrix™ [41].

Another promising stem cell derived from dental tissue is PDLSCs. A periodontal ligament is specialized connective tissue responsible for the regeneration of adjacent periodontal structures. PDLSCs are multipotent cells with high proliferation activity and the capability to differentiate into osteoblasts, cementoblasts, chondroblasts, and adipocytes [42]. Therefore, PDLSCs have also been examined as a possible source for bone regeneration. An in vivo study by Tour et al. (2012) demonstrated the ability of allogenic PDLSCs to form alveolar bone, periodontal ligament, and cementum-like tissue to repair periodontal defects caused by periodontitis [43]. More recently, Iwata et al. (2018) conducted a single-institute clinical study in which the authors created autologous three-layered PDLSC sheets combined with β-tricalcium phosphate bone fillers and transplanted them into the bony defects of 10 patients suffering from chronic periodontitis. The clinical outcomes were evaluated at 3 and 6 months after transplantation. A significant healing process of deep periodontal defects was detected in all cases, including an increase in radiographic bone height and reduction in periodontal probing depth without severe adverse effects. However, the main constraint of this study was the small number of patients. Nevertheless, the “Cell sheet engineering technology” developed by the authors is a promising approach that could be implemented in routine tissue regenerative techniques in dentistry [44].

A relatively newly discovered population of oral stem cells easily collected from surgically removed periapical cysts are hPCy-MSCs, which exhibit similar characteristics to other dental tissue-derived MSCs [45]. Tatullo et al. (2015) drew attention to the advantages of hPCy-MSCs in bone regeneration over the use of DPSCs. According to the qRT-PCR analyses performed, the hPCy-MSCs displayed higher potential to differentiate into osteoblast-like cells, whereas DPSCs tended to give rise to odontoblasts [46]. In the follow-up study, Tatullo et al. (2019) seeded hPCy-MSCs on PLA-based mineral-doped scaffolds to observe their proliferation, viability, and osteogenic/odontogenic gene expression. In all of the investigated parameters, the hPCy-MSCs displayed excellent results involving a high expression of odontogenic/osteogenic marker DMP-1 [47].

There are also several studies that compared bone healing capacities between various types of dental stem cells. For example, Nakajima et al. (2018) examined SHED cells’ osteoinductive potential and mineralization abilities and compared them with human DPSCs and human BM-MSCs. The stem cells seeded on a poly(lactic-co-glycolic acid) barrier membrane were transplanted to an artificial bone defect in the calvaria of an immunodeficient mouse. The histological analyses performed after 12 weeks post-transplantation revealed that SHED formed the largest osteoid area and synthesized more collagen fibers compared with other stem cell types [48]. Furthermore, according to comprehensive meta-analyses of preclinical studies focused on the therapeutic potential of five cell lineages (PDLSCs, BMSCs, DPSCs, GMSCs, and ADSCs) in periodontal tissue regeneration, PDLSCs and BMSCs were the most effective in new alveolar bone, cementum, and periodontal ligament formation [49]. Finally, in the most recent study, Qu et al. (2021) compared the osteogenic potential of four dental-derived MSCs, including DPSCs, PDLSCs, DFSCs, and alveolar bone-derived MSCs (ABMMSCs). Based on the analyses performed, such as osteogenic gene expression and alkaline phosphatase activity staining, ABMMSCs and PDLSCs exhibited higher osteogenic potential in alveolar bone regeneration [28].

Nonetheless, despite the abovementioned auspicious studies, there is undoubtedly a demand for future investigations focused on a better understanding of the biology of dental tissue-derived MSCs.

**Table 1:** Regenerative capacity of various dental tissue-derived mesenchymal stem cells (MSCs).

Type of Dental Tissue-Derived MSCs	In Vivo Models/Human Subjects	Site of Transplantation	Outcome	References
DPSCs	Immunocompromised mice	Dorsal surface	Generation of dentine/pulp-like structure	[27]
	Immunocompromised rats	Subcutaneous site of dorsal surface	Generation of bone tissue with an integral blood supply	[29]
	Immunocompromised mice	Subcutaneous site of dorsal surface	Maintenance of MSC characteristics; higher stability compared with PDLSCs in vivo	[7]
	6 patients aged 8 to 12 years old	Unilateral alveolar bone defect	Alveolar bone healing with no ectopic bone formation	[30]
paldSCs	30 patients	Alveolar bone defect	Improvement in vertical bone augmentation	[19]
DFSCs	Immunocompromised rats	Critical-sized calvarial defects	New bone formation	[35]
GMSCs	C57BL/6J mice	Second maxillary molar	Reduction in alveolar bone loss and new bone formation	[39]
	Athymic rodent models	Maxillary alveolar bone defect	Enhanced bone regeneration	[40]
PDLSCs	Immunocompromised rats	Calvarial critical-sized defect	Improvement of bone repair	[42]
	10 patients with chronic periodontitis	Root surface of defect site	Healing of deep periodontal defects	[43]
SHED cells	Immunocompromised mice	Calvarial artificial bone defect	Formation of osteoid	[47]
SCAP	Minipig model of periodontitis	Local injection in the site of defects	Increased alveolar bone and periodontal tissue regeneration	[23]

## 3. Osteogenic Potential of iPSCs in Dental Tissue Regeneration

iPSCs represent a significant breakthrough in the field of regenerative medicine as well as a rising option for cell-based therapy in dentistry involving alveolar bone regeneration. iPSCs are derived from patient’s somatic cells preventing immune rejection and can differentiate into several different cell lineages. Thanks to the abovementioned advantages, iPSC technology may be implemented as an alternative to autologous grafting, by which the patient-specific somatic cells are reprogramed into MSCs/osteoprogenitor cells, and seeded on an appropriate scaffold, and treated with bioactive molecules [50]. It is assumed that, in the case of dental tissue regeneration, the generation of iPSCs from dental tissue may be more beneficial than other tissue sources because of probable epigenetic memory maintenance of the source tissue [9,51]. In comparison with dental tissue-derived stem cells, dental tissue-derived iPSCs are more proliferative in vitro and its regenerative ability can be more easily reproduced [52]. Numerous studies have reported the successful generation of iPSCs from various types of dental tissues, such as exfoliated deciduous teeth [53,54,55], extracted wisdom teeth [56], oral mucosa fibroblasts [57,58,59], gingival tissue [60,61,62,63,64], periodontal ligament [65], and the dental pulp [66,67,68].

It has been shown that iPSCs could be easily differentiated into MSCs (iPSC-MSCs) with conspicuous benefits over direct differentiation of iPSCs into osteoblasts, involving reduced risk of tumor formation and higher predisposition of iPSC-MSCs to osteogenic differentiation. iPSC-MSCs retain almost the same osteogenic potential as MSCs derived from other sources, such as bone marrow, umbilical cord, adipose tissue, etc. [69]. Moreover, obtaining iPSC-MSCs from dental tissue is a much less invasive and cost-effective method requiring fewer steps during cultivation. Several studies showed that iPSC-MSCs could promote the repair of periodontal defects by increasing bone tissue regeneration and mineralization of newly formed bone [70,71,72,73].

## 4. Osteogenic Differentiation of iPSCs

For the osteogenic differentiation of iPSCs, several protocols based on differentiation protocols for MSCs have been developed. A more detailed insight into osteogenic differentiation strategies is discussed in our previous work [74]. In general, the generation of osteoprogenitor cells from iPSCs can be achieved by the direct differentiation of iPSCs through embryoid body (EB) formation into bone progenitors or through the differentiation of iPSCs into iPSC-MSCs followed by their final osteogenic differentiation [9]. Apart from traditional methods, Zhong et al. (2019) highlighted the role of conditioned media obtained from osteoblast cultures in inducing the differentiation of iPSC-MSCs into osteogenic lineages [69]. Furthermore, the osteogenic differentiation process involves the application of osteoinductive cultivation media with bioactive molecules such as ascorbic acid, b-glycerophosphate, dexamethasone, bone morphogenetic proteins (BMPs), insulin-like growth factor 1 (IGF-1), fibroblast growth factor -2 (FGF-2) retinoic acid, and vitamin D3 [75,76,77].

BMPs (BMP-2 and BMP-6) play main roles in the differentiation process thanks to their ability to enhance the proliferation and differentiation of MSCs or osteoprogenitor cells by activating the expression of genes involved in bone formation [72,78,79]. The first evidence of antibody-mediated osseous regeneration (AMOR) using anti-BMP2 antibodies (Abs) for the osteogenic differentiation of iPSC-MSCs was reported by Wu et al. (2018). This concept is based on capturing endogenous osteogenic BMPs in situ; therefore, it bears several advantages over direct exogenous BMP2 administration, such as higher safety, better efficacy, and faster endogenous regeneration [80]. More recently, Song et al. (2021) investigated the osteoinductive effect of the synthetic inorganic molecule SB431542, which enhances the differentiation of iPSCs into MSCs followed by further activation of BMP signaling of the TGF superfamily. This study demonstrated that the combination of SB431542 with calcium phosphate cement scaffold greatly enhanced proliferation, osteogenic differentiation, and bone mineral synthesis of iPSC-MSCs. More than that, the authors did not observe any cytotoxic effect of SB431542, making this compound a possible alternative to the quite expensive BMP-2. Nonetheless, there is a necessity for further research on in vivo models [81].

A combination of small molecules as osteogenic inducers incorporated into a xeno-free (E8/VTN) strategy was authored by Zujur et al. (2020). iPSCs were gradually cultivated in media with different types of small molecules, including CHIR99021, cyclopamine, helioxanthin derivative TH, smoothened agonist SAG, FGF2, and additional supplements. After 21 days of cultivation, mature osteoblasts were detected. Furthermore, the osteoinductive effect of this protocol was refined by its combination with a 3D scaffold to establish a xeno-free 3D osteogenic system [82].

Another recent study published by Li et al. (2021) evaluated the effect of distal-less homeobox 3 (DLX3) on the proliferation and osteogenic differentiation of iPSC-MSCs. iPSC-MSCs were transfected with DLX3 overexpression plasmids, and the expression of osteogenesis-related markers and proteins was analyzed and compared with the control group. After seven days post-transfection, the RT-qPCR results showed an evident increase in alkaline phosphatase (Alp), osteopontin, osteocalcin, and collagen type 1 (Col-1) expression. Besides that, Alp staining and mineralized nodule counting revealed a significantly higher number of mineralized nodules in iPSC-MSC-DLX3 over the control group. Taken together, this study demonstrated the positive effect of DLX3 on osteogenic differentiation; however, the exact mechanisms of its action have not been fully understood yet [83]. Another recently found enhancer of osteogenic differentiation is menaquinone-7 (MK-7), in which a positive effect was proven on iPSC-MSCs, exhibiting a notable increase in Runx-2 expression, Alp activity, and collagen deposition [84].

In summary, the osteogenic induction of iPSCs is a complex process. Its success depends on the origin of iPSCs, reprogramming method, type of scaffold, differentiation method, and composition of cultivation media. On account of the mentioned criteria, more research has yet to be conducted to find an ideal way to differentiate iPSCs into a safe and viable osteogenic cell population.

## 5. Scaffolds Suitable for Alveolar Bone Regeneration

The fundamental component of bone tissue engineering, which significantly improves available treatment options, is the establishment of 3D stem cell culture on a “cell-friendly” biodegradable scaffold enriched by bone-forming factors. Nowadays, several types of stem cells (MSCs/iPSCs/bone progenitor cells) are already being used in combination with biomaterials that guide them in osteogenic differentiation, resulting in final bone graft generation.

The composition of a scaffold material is critical for proper bone tissue regeneration; thus, it needs to meet the following criteria: appropriate ability to retain cells, biocompatibility, safety, non-toxicity, osteoinduction, osteoconduction, and biodegradation. In other words, the most suitable material should be osteoinductive, should hold its content placed at the site of the bone defect, and should break down safely without any toxicity. Moreover, the scaffold must be shapable into different forms with optimal porosity to fill the bone defect. Some materials with the abovementioned properties include the combinations of bioceramics (hydroxyapatite, calcium-phosphates, bioactive glasses, and calcium sulfate), natural polymers (collagen, coralline, chitosan, pectin, silk, hyaluronic acid, and alginate), and synthetic polymers (polylactic acid (PLA), polyglycolic acid (PGA), polylactic-co-glycolic acid (PLGA), polyamide polycaprolactone (PCL), and decellularized extracellular matrix (dECM)) [4,85,86]. Equally important is the accurate fabrication of the scaffold, which can now be easily achieved through novel 3D bioprinting technologies. Moreover, the combination of 3D-bioprinted scaffolds with cell-based therapy allows biocompatible materials and cells to be placed in the exact position in 3D space and enables the precise delivery of bioactive molecules with osteoinductive effects, such as BMP4, FGF2, IGF1, and PDGF [87].

The following paragraph mentions several interesting studies concerning the generation of novel scaffold materials combined with stem cells to reach maximal osteogenic properties suitable for alveolar bone regeneration (Table 2).

Duan et al. (2011) investigated for the first time the benefits of iPSCs and enamel matrix derivatives (EMDs) in periodontal tissue regeneration. First, the RT-PCR analyses showed that the combination of apatite-coated silk scaffolds, EMD gel, and iPSCs significantly increased the mRNA expression of Runx-2, which is the main transcription factor of osteogenic differentiation promoting the differentiation of MSCs into preosteoblasts. Second, the iPSCs combined with EMDs were transplanted into the periodontal fenestration defect of nude mice models and examined 24 days post-surgery. According to histological and micro-CT analyses, a new bone tissue, which filled almost the whole area of bone injury, was observed [70].

A scaffold with high osteoinductive properties is macroporous calcium phosphate cement (CPC), which is a nano-mineral bone cement with load-bearing ability, bioactivity, and better affinity for cell seeding. Liu et al. (2013) used a CPC scaffold biofunctionalized with Arg–Gly–Asp (RGD-CPC), in which BMP2 gene-modified iPSC-MSCs were seeded. The RT-PCR analyses and histological analyses performed on days 14 and 21 showed higher efficacy of osteogenic differentiation followed by bone matrix mineralization compared with the control group, indicating that the use of RGD-CPC material in bone engineering is beneficial [88].

The research group of Lin et al. (2019) reviewed different combinations of seeded cells (bi-culture and tri-culture) on macroporous RGD-CPC scaffolds. According to the investigation, the seeding of the tri-culture composed of iPSC-MSCs, endothelial cells, and pericytes accomplished increased pre-vascularization of scaffolds in vitro and new bone formation accompanied by vascularization in vivo compared with a monoculture [73]. However, according to another review, using small animal models such as rats or mice is not sufficient for in vivo experiments; therefore, to prove such a high osteoinductive effect of this construct, further investigation of osteogenesis and angiogenesis on larger animals is inevitable [89].

It is important to mention the significant role of bioactive glasses (BGs) in hard tissue reconstruction such as bone and teeth. BGs have an outstanding ability to form direct bonds to bone, and their ionic products dissolved in body fluid stimulate osteoblast proliferation and angiogenesis. In addition, a combination of BGs with trace elements, such as silver, lithium, copper, cobalt, and zinc enhances their positive effect on bone regeneration [90,91]. For instance, 3D copper-containing mesoporous BGs are considered multifunctional biomaterials used in bone reconstruction [92]. Zhang et al. (2018), in their recent study, constructed novel 3D-printed BG block/chitosan nanoparticle composites loaded with BM-MSCs to study their osteogenic activity in bone defect reconstruction. The tissue-engineered bone composites were implanted into alveolar defects of rhesus monkeys. After 12 weeks post-transplantation, various analyses revealed the formation of new alveolar bone tissue, which was remarkably close to the normal bone in mass, structure, and density [93].

To find suitable scaffold materials for alveolar bone regeneration, Trivedi et al. (2020) examined the abilities of hydroxyapatite-collagen (HA-Col) scaffolds to enhance proliferation and osteogenic differentiation of DPSCs. According to the results of a sulforhodamine colorimetric assay, an alkaline phosphatase assay, and scanning electron microscopy (SEM), the HA-Col scaffold supported DPSC attachment and created a microenvironment that augmented the differentiation of DPSCs into osteogenic cells. Therefore, the researchers suggested that the HA-Col scaffold may represent an optimal material for alveolar bone defect reconstruction [94].

Three-dimensional collagen matrices have also proved to be a reliable scaffold material for rebuilding lost tissue within alveolar bone defects. A short time ago, the beneficial effect of such scaffold constructs in future periodontal surgery was investigated by Lin et al. (2021). The authors focused on the adhesive, migratory, proliferative, and differentiation potentials of MSCs and pre-osteoblastic cells seeded on four 3D collagen-based matrices: dried acellular dermal matrix, hydrated acellular dermal matrix, non-crosslinked collagen matrix, and crosslinked collagen matrix. As expected, the results showed magnified motility of the osteoprogenitor cells in all matrices, though the best outcomes in terms of growth and osteogenic differentiation of progenitor cells was observed in a hydrated acellular dermal matrix and crosslinked collagen matrix, demonstrated by the significant expression of osteogenic differentiation markers (Runx-2, Alpl, Dlx5, Ibsp, Bglap2, and Phex). In addition, 3D collagen scaffolds were pre-coated with EMD and recombinant BMP-2 to enhance osteogenic differentiation. Further steps should focus on the in vivo investigation of this indisputable positive outcome [95].

A new approach was published by Chien et al. (2018), who developed a 3D iPSC-BMP-6-hydrogel complex with thermosensitive properties and injected it into murine models of maxillary-molar defects. Micro-CT analyses performed within six weeks post-implantation revealed an extensive amount of newly formed bone tissue within periodontal defects, proving the high osteogenic capability of iPSCs combined with BMP-6 [72].

Graphene-based nanomaterials also belong to the increasingly used types of scaffold materials in dental tissue engineering. Concerning osteogenesis, it was found out that graphene-coated scaffolds support the proliferation of MSCs and enhance osteogenic differentiation [96]. Park et al. (2021) came up with an innovative approach using the 3D bioprinting method for PCL scaffold fabrication. Such 3D-printed scaffolds were additionally treated with oxygen plasma and coated with graphene oxide (GO). The authors decided to use GO because of its osteoinductive properties and hydrophilicity since the PCL itself possesses strong hydrophobicity impeding cellular adherence. The treatment with oxygen plasma was performed to further improve the coating efficacy. The cell cultures seeded on scaffolds were PDLSCs, which have favorable capabilities to induce the repair of alveolar bone defects. The results from various analyses focused on osteoinductivity, and osteogenic differentiation proved the great potential of GO-coated 3D-printed PCL scaffolds for alveolar bone regeneration [97].

**Table 2:** Selection of novel studies using innovative biomaterials combined with stem cells for bone tissue engineering.

Scaffold Material	Seeded Cells	Outcome	References
Apatite-coated silk scaffolds + EMD gel	iPSCs	Significant expression of Runx2; new bone tissue formation in vivo	[70]
RGD-CPC	iPSC-MSCs	Higher efficacy of osteogenic differentiation and bone matrix mineralization	[88]
RGD-CPC	iPSC-MSCs; + endothelial cells + pericytes	Increased scaffold pre-vascularization in vitro; new bone formation and vascularization in vivo	[73]
3D-printed BG block/chitosan nanoparticles composites	BM-MSCs	New alveolar bone tissue formation in vivo	[93]
HA-Col	DPSCs	Supported attachment of DPSCs and formation of microenvironment for osteogenic differentiation in vitro	[94]
3D collagen-based matrices + EDM + BMP-2	MSCs	Significant expression of osteogenic markers; enhanced osteogenic differentiation in vitro	[95]
3D BMP-6-hydrogel complex	iPSCs	new bone tissue formation in vivo	[72]
Graphene oxide-coated 3D-printed PCL scaffold	PDLSCs	Enhanced osteoinductivity and osteogenic differentiation in vitro	[97]

## 6. Extracellular Vesicles—New Therapeutic Agents in Bone Regeneration

It is well known that extracellular vesicles (EV) such as exosomes and microvesicles are key paracrine effectors that participate in maintaining cell or tissue homeostasis through cell-to-cell communication. Exosomes—nanosized (30–150 nm) membrane-bound EVs—are secreted into the extracellular fluid by most cells through fusion with the cytoplasmic membrane; afterwards, to deliver their content to a target cell, they bind to its surface directly or via specific ligands [98]. The cargo carried by exosomes influences crucial cellular processes of targeted cells, including apoptosis, proliferation, migration, and specific differentiation. Concretely, exosomes contain proteins, lipids, mRNA, miRNAs, and lncRNAs, while the most abundant components are miRNAs, which regulate gene expression of target cells. It was further demonstrated that the miRNA content is highly specific to the donor cell type and cell conditions. Importantly, it was found out that miR-21 promotes angiogenesis both in vivo and in vitro. Some of the most common proteins include a group of scaffolding membrane proteins, including CD63, CD81, and CD9, which serve as surface-specific markers [99,100,101]. The standard method for exosome isolation is differential ultracentrifugation, which is usually performed in conjunction with iodixanol or sucrose cushions to reach higher purity. Other methods for exosome purification are immunoaffinity chromatography or size exclusion chromatography [98,102].

In the field of dentistry, the MSCs derived from oral tissue represent the most favorable cell type for exosome harvesting. Thanks to the high content of bioactive molecules, exosomes display similar activity to MSCs; moreover, they reduce the inherent safety risk, thus representing a cell-free alternative in bone tissue engineering and regeneration [103,104]. Furthermore, the therapeutic use of exosomes eliminates the risk of tumor formation because they do not mutate, do not duplicate, and cannot initiate metastasis. Recently, it was demonstrated that their immunosuppressive, immunomodulatory, and regenerative properties could be further increased by preconditioning MSCs [105].

The osteoinductive influence of exosomes derived from MSCs (MSC-Exos) has been published in several studies, while in most of them, the transplantation of MSC-Exos enhanced osteogenesis, angiogenesis, and bone regeneration [100,101,106,107,108,109,110,111]. Similarly, with the description of iPSCs, protocols focused on bone regeneration based on exosomes obtained from iPSCs (iPSC-Exos) have taken the stage [112,113,114] (Table 3).

It is necessary to point out that one of the crucial components for the successful outcome of exosome-derived bone regeneration is the selection of proper carrier material to load exosomes and to release them in the target cell site of injury. It is assumed that hydrogels could meet these criteria considering their ability to stabilize and retain exosomes while maintaining the stability of exosomal cargo [115]. The task of constructing an “exosome-friendly” material was accomplished by Zhang et al. (2021), who encapsulated MSC-Exos in hyaluronic acid hydrogel and incorporated them with nanohydroxyapatite/poly-ε-caprolactone (nHP) 3D-printed scaffolds. In vitro as well as in vivo analyses of this composite revealed accelerated osteogenesis and angiogenesis with the ability to repair large cranial bone defects in rats [101].

All in all, according to recent studies, the role of exosomes in bone tissue regeneration is indisputable, offering a cell-free alternative to conventional strategies. However, the use of “Exo-therapy” in a clinical setting is still restricted due to the lack of generally accepted procedures for their isolation, separation, delivery, storage, and standardization of the most favorable therapeutic dose.

## 7. Conclusions and Future Perspectives

Bone tissue engineering techniques together with cell-based therapy have considerably progressed during the past few years and represent a remarkable opportunity for hard dental tissue reconstruction to avoid more invasive and less predictable procedures such as autologous bone grafts. To achieve a successful outcome in alveolar bone regeneration, an ideal synergy between the source of stem cells, osteoinductive molecules, and scaffold materials is inevitable. However, as concluded by Kargozar et al. in their comprehensive review, the main question regarding the ideal cell source for bone tissue engineering has not been solved yet. The mentioned cell lines with osteoinductive properties possess more or less issues such as restricted availability, prolonged handling time, and safety risks [116].

Some suitable cell lineages that can be effectively differentiated into osteogenic cells include dental tissue-derived stem cells and iPSCs. Particularly, iPSCs represent an unlimited patient-specific source of stem cells, providing high biocompatibility with almost no risk of immune rejection. The same importance is placed on selecting 3D biocompatible scaffold materials that can mimic the bone composition and microstructural organization as closely as possible. This goal could be achieved by the development of novel technologies based on 3D bioprinting of nanomaterials, such as graphene-based nanomaterials [96]. Finally, yet importantly, incorporating bioactive molecules, such as growth factors, has a significant impact on bone regeneration efficacy.

Exosomes, the new players in the field of regenerative medicine, have garnered much attention as possible cell-free therapeutic agents minimizing safety risks related to cell transplantation. However, there is a need to establish standards for their purification and quality control to accelerate their clinical applications in oral regenerative therapy.

It is undeniable that research in bone regenerative medicine holds great potential for translation into clinical practice, which has been already proved by numerous clinical studies. However, it is necessary to develop more efficient and safe protocols for cell reprogramming, osteogenic differentiation, precise cell purification, scaffold fabrication, and final transplantation.

## Figures and Tables

**Table 3:** MSC-/induced pluripotent stem cell (iPSC)-derived exosomes (Exos) in bone regeneration.

Exosome Source	Isolation Method	Outcome	References
BM-MSCs	Ultracentrifugation of BM-MSC-conditioned media	MSC-Exos facilitated femur fracture healing in mice	[105]
iPSC-MSCs	Ultracentrifugation of iPSC-MSC-conditioned media	iPSC-MSC-Exos efficaciously stimulated bone regeneration and angiogenesis in critical-sized calvarial defects in rats	[111]
iPSC-MSCs	Ultracentrifugation of iPSC-MSC-conditioned media	iPSC-MSC-Exos significantly prevented osteonecrosis and increased microvessel density in femoral head	[113]
ADSCs	Ultracentrifugation of ADSC-conditioned media	ADSC-Exos increased bone formation in critical-sized mice calvarial defects	[107]
Umbilical MSCs treated under hypoxic condition	Ultracentrifugation of media with sucrose/D2O cushion conjunction	Hypo-exosomes promoted femoral fracture healing by transferring miR-126 in mice	[99]
BM-MSCs	Ultracentrifugation of BM-MSC-conditioned media	Osteogenesis, angiogenesis, and bone healing in a fracture model of rat femoral nonunion	[107]
hDPSCs	Ultracentrifugation of hDPSC-conditioned media	hDPSC-Exos facilitated osteogenic differentiation of BM-MSCs; mice calvarial defect repair by hDPSC-Exo loaded constructs	[108]
BM-MSCs	Ultracentrifugation of BM-MSC-conditioned media	MSC-Exos promoted angiogenesis and osteogenesis in vitro; restoration of bone formation and mechanical quality in vivo	[109]
Umbilical MSCs	Ultracentrifugation of umbilical MSC-conditioned media	MSC-Exos seeded on 3D hydrogel scaffold promoted the repair of cranial defects in rats	[100]
